# Prenatal Diagnosis of Granular Cell Tumor

**Published:** 2014-03

**Authors:** Maliheh Kadivar, Razieh Sangsari, Azin Alavi

**Affiliations:** 1 Department of Neonatal Care Unit, Children’s Hospital Medical Center, Tehran University of Medical Sciences, Tehran, Iran;; 2 Department of Obstetrics and Gynecology, Shariati Hospital, Hormozgan University of Medical Sciences, Hormozgan, Iran

**Keywords:** Gingiva, Granular cell tumor, Newborn, Prenatal diagnosis

## Abstract

Congenital granular cell tumor (GCT) is a relatively rare intraoral benign tumor, approximately 200 cases of which have been reported in the neonatal period worldwide. The newborn infant may have feeding problems and respiratory difficulties due to airway obstruction. This lesion may be diagnosed by prenatal ultrasonography and simple resection is mostly required. We report a case of an adult type of GCT in a newly born infant, who presented with an intraoral protruding mass with a prenatal diagnosis. This article describes the prenatal course, clinical, and pathological characteristics, and management of the GCT.

## Introduction

Tumors of the oral cavity, albeit not common in the neonatal period, may cause feeding problems and airway obstruction, leading to emergency situations after birth with difficult airway management.^1^ These lesions also may protrude from the baby’s mouth with a monstrous appearance.^2^^,^^3^

Granular cell tumor (GCT) is a relatively rare tumor and is almost always benign. The most frequent locations are the tongue, skin, and soft tissue. The tumor typically develops in adults in the third and sixth decade of life and its occurrence in neonates is extremely rare.^4^One of these rare tumors is congenital GCT, which could be diagnosed prenatally.^2^^,^^5^^,^^6^

Congenital GCT grows only in utero, especially during the 3rd trimester of gestation.^1^^,^^7^ The histogenesis of this tumor has yet to be clearly defined, but various origins such as gingival endothelial, mesenchymal, mioblastic, odontogenic, neurogenic, fibroblastic, and histocytotic have been proposed as possibilities.^2^^,^^6^Because of female predominance, the influence of maternal estrogen and fetal ovarian hormones has been postulated in the pathogenesis of the tumor, with spontaneous regression after maternal estrogen withdrawal.^8^This possible influence of estrogen and progesterone receptors has been investigated through immunohistochemical studies.^8^ The tumor arises more commonly from the maxilla than the mandible with a 2:1 ratio,^6^with the involvement of both maxilla and mandible in 10% of cases.^1^^,^^3^^,^^7^ The typical location is the anterior alveolar ridge of the maxilla.^3^^,^^7^^,^^8^The GCT is usually single, but multiple tumors also have been  reported.^8^ The lesion may be sessile or pedunculated with pink, firm consistency and a smooth lobulated surface from a few millimeters to 9 cm.^2^^,^^6^^,^^8^

We report a case of GCT in a female newborn, who presented in the prenatal period with an intraoral mass which was protruding from her mouth and was not congenital. We describe the prenatal course as well as the clinical and histological characteristics of the lesion and its management. 

## Case Presentation

**Figure 1:**
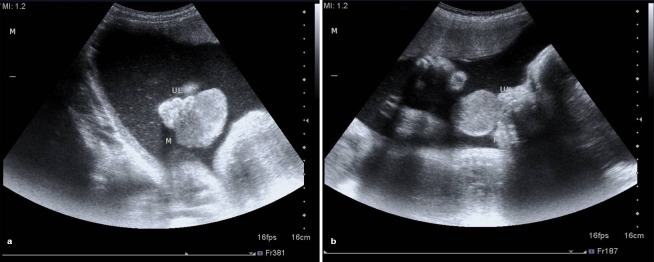
Sonography at 35 weeks of gestation, showing a well-defined, lobulated and hypoechoic mass protruding from the mouth of the fetus in axial (a) and sagittal (b) views.

**Figure 2:**
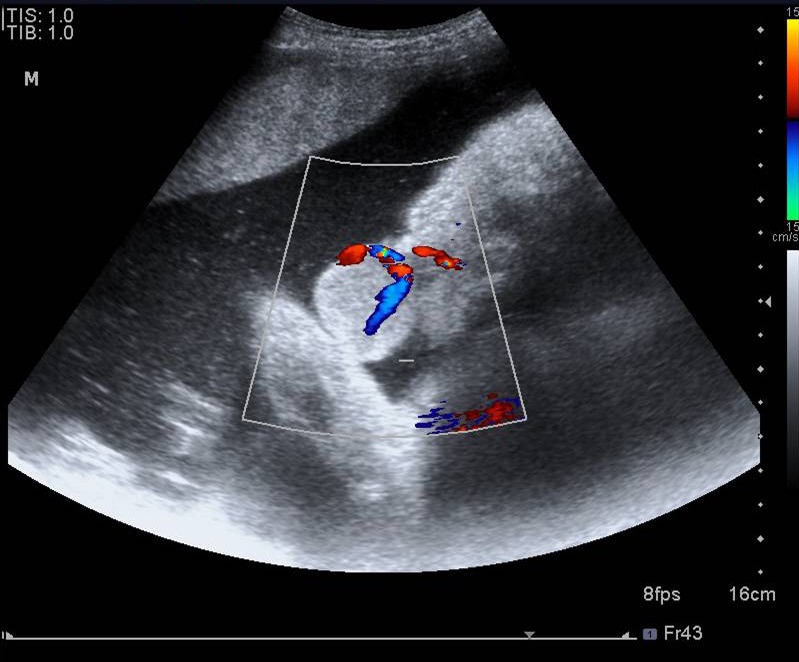
Ultrasound evaluation with color Doppler, showing a mass protruding from the mouth with a branching pattern of the feeder vessels.

**Figure 3:**
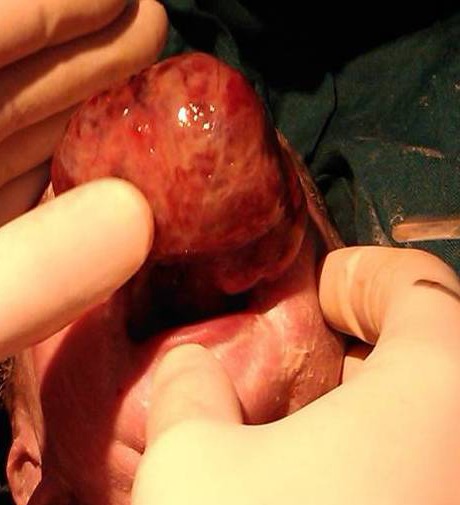
Appearance of the mass in the oral cavity in the delivery room and the location on the maxillary alveolar ridge.

**Figure 4:**
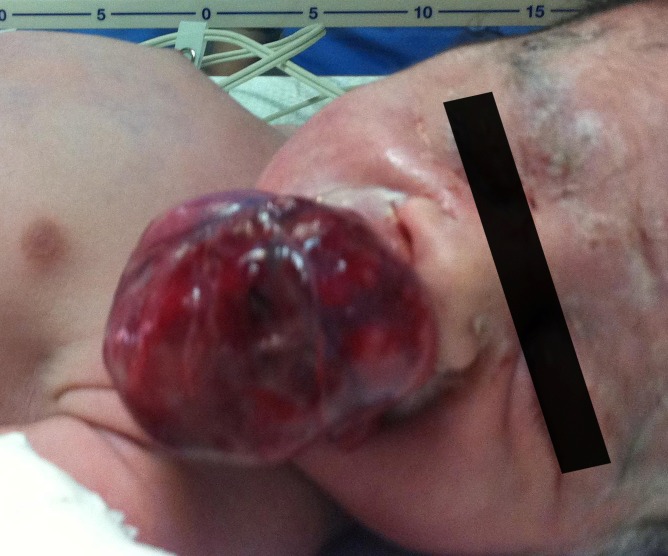
Appearance of the baby with the oral mass at the time of admission in the Intensive Care Unit.

A newborn female infant was transferred to the Neonatal Intensive Care Unit of Children’s Hospital Medical Center of Tehran from a maternity hospital shortly after birth on 31^st^ July, 2011 because of an intraoral mass. She was a product of Cesarean section because of transverse lie presentation at 38 weeks of gestation with a birth weight of 3150 g, head circumference of 35 cm, height of 50 cm, and APGAR scores of 9 and 10 at one and 5 minutes, respectively. Her mother, a 20-year-old primiparous woman, was referred to the prenatal clinic of another hospital due to a congenital oral mass, which was diagnosed in the 35th week of gestation (figure 1). She had a normal previous ultrasound. The primary prenatal impression of this hyperechoic lobular mass from the upper part of the mouth was congenital teratoma, which was measured to be about 42×35×29 mm by ultrasound (figure 1). Ultrasound evaluation with color Doppler showed a mass protruding from the mouth with a branching pattern of the feeder vessels (figure 2). She had no history of either medication during pregnancy or hereditary diseases or oral masses in other members of her family. The prenatal ultrasound also revealed mild polyhydramnios (AFI=26) with a normal color. She was pink and vigorous after birth without any signs of respiratory distress or evidence of airway obstruction, even though she had a mass in the oral cavity (figure 3). Clinical examination at the time of admission revealed a pedunculated irregular mass, approximately 60×30×45 mm in size, attached to the gingiva of the anterior alveolar ridge of the maxilla in the midline of the oral cavity (figure 4). She had no other abnormalities on physical examination. Paraclinical studies did not reveal any other abnormalities. The serum alfa-fetoprotein level was 17300 ng/mL, which was within normal range for age. Facial CT-scan demonstrated a soft tissue mass, 62×33 mm in size, extending from the hard palate without any connection to the bone or the nasal cavity.

After receiving informed verbal consent from the baby's parents, pediatric surgical and otolaryngological consultation was done. The intraoral mass was completely resected at second day of life. The baby was intubated and mechanically ventilated for a day after surgery for proper healing of the oral cavity and further pain management. Breastfeeding was started at 4^th^ day of life, and the baby tolerated it without any problems.

**Figure 5:**
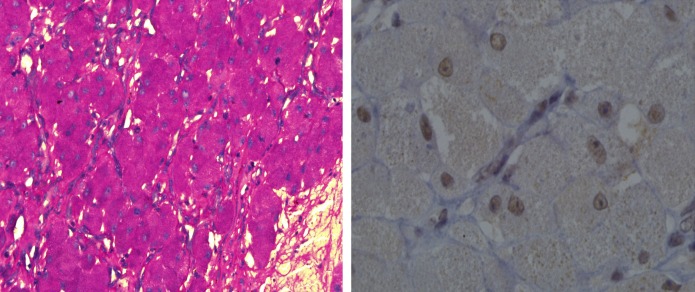
Microscopic appearance and PAS, showing intense positivity in the cytoplasm of the tumor cells.

The pathologic examination of the removed tissue revealed an ovoid creamy to grayish tumor with an irregular and lobulated smooth surface. It was homogenous cream-gray, with fine lobulation on the cut section. The microscopic examination showed homogenous solid sheets of monomorphic large polygonal cells with eccentric small round nuclei and an eosinophilic granular cytoplasm (figure 5). In the stroma, there was a delicate network of blood vessels. S100 immunostaining showed nuclear reactivity as well as fine cytoplasmic staining X1000. Therefore, the definite diagnosis of an adult type of GCT^3^was confirmed and congenital epulis was ruled out.

The baby was discharged at 6th day of life with regular check-ups in the following 12 months, which revealed no evidence of recurrence.

## Discussion

GCT is also known as Abrikossoff's tumor, granular cell nerve sheath tumor,^4^ granular cell schwannoma,^9^ and granular cell myoblastoma.^6^^,^^9^ The classic location of this tumor is the tongue;^9^it has been seen, however, in innumerable other locations such as the skin, vulva, breast, larynx, bronchus, esophagus, and stomach.^9^ The GCT may occur at any age,^9^ from 11 months to 104 years old,^5^but most frequently in the 4th to 6th decade of life.^4^ This tumor is usually small, although there have been reports of cases measuring up to 5 cm in diameter.^9^

The congenital GCT has also been reported. This tumor, also known as congenital epulis, is a rare gingival neoplasm that affects the alveolar ridge in the newly born. The congenital form is mainly located in the gingival of the maxillary alveolar ridge,^6^^,^^10^ with a strong female  predominance (9:1).^6^

As long as the GCT has a benign clinical course, the incidence of local recurrence is 5-10% after surgical resection^2^^,^^9^and it can rarely undergo malignant transformation.^2^^,^^9^By contrast, the congenital GCT has a benign clinical course and does not show malignant degeneration and recurrence, even with incomplete resection.^2^^,^^6^

Cytologic smear of the GCT is moderately to highly cellular, with cells in loosely arranged groups or lying singly in a granular background. Most cells are oval shaped, with relatively uniform nuclei with small nucleoli and moderate to abundant amounts of fragile granular cytoplasm. While the adult form of the GCT^2^ is strongly and diffusely positive for S100, which is specific for Schwann cells, the congenital GCT is negative for S100^2^^,^^11^ In the congenital form, the prenatal diagnosis by ultrasound has been reported mostly in the third trimester of pregnancy.^2^ It allows prenatal counseling with parents, referring for delivery to a well-equipped center with pediatric surgeons, and preparation of an expert team in the delivery room for potential airway obstruction.^2^^,^^5^ Because of the obstruction of the fetal oral cavity and the amniotic fluid, swallowing inability and polyhydramnios may occur^2^ Other oropharyngeal masses such as teratoma, lymphangioma, hemangioma, and neurofibroma should be included in the differential diagnosis.^2^^,^^8^

We herein described a female newborn infant who had a prenatal diagnosis of an intraoral mass on ultrasonography. The prenatal sonographic imaging revealed a well-defined lobulated mass protruding from the mouth and with a branching pattern of the feeder vessels. The distinctly crowded branching vessel in the GCT is in contrast to the disorganized vascularization with a high flow seen in oral hemangioma.^2^^,^^8^After the complete removal of the tumor, microscopic examination demonstrated a granular cell tumor which had nuclear reactivity as well as fine cytoplasmic staining according to  S100 immunostaining, suggesting an adult form of the GCT* .^2^*

## Conclusion

While an intraoral mass can be diagnosed by prenatal utrasonography, confirmation is only possible histologically after birth. The multidisciplinary team approach is important in the management of these cases. When there is a congenital mass in the oropharyngeal region, the possibility of the presence of the GCT should be contemplated. 
